# Rare case of aspergillus brain abscess in an immunocompromised patient

**DOI:** 10.1002/ccr3.6169

**Published:** 2022-08-18

**Authors:** Seyed Parsa Eftekhar, Roghayeh Akbari

**Affiliations:** ^1^ School of Medicine Babol University of Medical Sciences Babol Iran; ^2^ Department of Nephrology Babol University of Medical Sciences Babol Iran

**Keywords:** aspergillus, brain abscess, immunosuppression, infection, kidney transplantation, neuronavigation surgery

## Abstract

We present a case of aspergillus brain abscess in a 48‐year‐old woman with a history of kidney transplantation and no underlying central nervous system (CNS) disease. Follow‐up of the patient for 4 years shows normal findings. Early diagnosis and aggressive treatment could improve the prognosis of this fatal complication.

## Graphical abstract

Early diagnosis and aggressive antifungal therapy accompanied by neuronavigation‐guided surgery can increase the chance of recovery in immunosuppressed patients with aspergillus brain abscess.

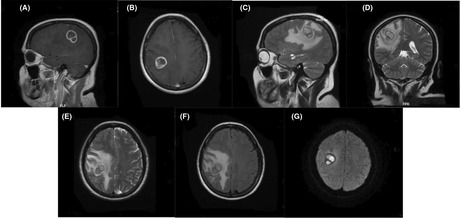

## 1 INTRODUCTION

Increasing numbers of chronic kidney disease (CKD) concerns society.^1^ Different therapies may slow the progression of the disease. However, many patients finally develop the end‐stage renal disease (ESRD). Kidney transplantation is the choice treatment for ESRD, which improves patients' prognosis and quality of life. Despite the advances in transplantation therapy, several life‐threatening complications may occur.

Complications involve many organs and could be infectious (like opportunistic infections) or non‐infectious (such as hematoma, perinephric fluid collection, acute tubular necrosis, and rejection).^2^ Although immunosuppression therapy reduces the risk of graft rejection, it increases the risk of infection, the second cause of morbidity and mortality in these patients.^2^

Post‐transplantation infections are predictable, and it depends on the timing after transplantation. Opportunistic infections such as aspergillus, atypical molds, and mucor species usually occur after 5 months.^3^ Although infection is the most common complication of the CNS after renal transplantation, brain abscess is a rare consequence.^4^ Despite significant advances in the field of transplantation, complications are inevitable. The current study presents an aspergillus brain abscess secondary to kidney transplantation in a 48‐year‐old woman, a rare and fatal complication of kidney transplantation.

## 2 CASE PRESENTATION

A 48‐year‐old woman was admitted with a persistent headache. The patient had a history of renal transplantation about 10 months before the onset of symptoms. Moreover, she had diabetes mellitus type 1 and was receiving insulin. She had a headache for 2 months, accompanied by concurrent photophobia, hemiparesis, and paresthesia of the left upper limb. The headache is initiated in the occipital region and shifts to the frontal region in a few moments. Moreover, the Barthel index (a scale measuring performance in daily activities^5^) was 14/20. The patient's immunosuppression regimen included cyclosporine (100 mg twice daily), tacrolimus (1.5 mg twice daily), mycophenolic acid (720 mg twice daily), and prednisolone tablets (5 mg daily) (blood level of cyclosporine and tacrolimus have been shown in Table 1).

**TABLE 1:** Laboratory findings

Parameter	Time of admission	2 months after brain surgery	4‐years follow‐up	Normal value
Urine				
pH	6	6	5	4.6–8.0
Protein	Negative	Negative	Negative	
Color	Yellow	Yellow	Yellow	
Blood	Negative	Negative	Negative	
Urine culture	Negative	Negative	Negative	
Blood				
White blood cells (/μl)	10,400	10,110	10,100	4400–11,000
Red blood cells ($10^{6}$/μl)	4.10	5.0	4.33	4.5–5.1
Hemoglobin (g/dl)	11.1	13.2	11.7	12.3–15.3
Platelet ($10^{4}$/μl)	25.6	28.5	23.3	15.0–36.0
AST (U/L)	12	11	19	<31
ALT (U/L)	17	23	15	<31
Uric acid (mg/dl)	6.1	8	6.4	1.9–7.9
Bilirubin Total (mg/dl)	0.5	0.5	0.6	0.1–1.2
Bilirubin Direct (mg/dl)	0.1	0.1	0.15	<0.3
Alkaline phosphatase (U/L)	226	224	230	64–306
Calcium (mg/dl)	9	9.5	10.3	8.6–10.3
Sodium (mmol/L)	140	140	138	136–146
Potassium (mmol/L)	4.10	4.60	4.44	3.8–5.1
Total protein (g/dl)	6.80	6.70	7	6.0–8.3
Serum albumin (g/dl)	4.60	4.65	4.85	3.4–5.4
Blood urea nitrogen (mg/dl)	15	16	72	5–22
Creatinine (mg/dl)	1.1	1.02	2.27	0.6–1.1
Fasting plasma glucose (mg/dl)	79	84	90	70–100
C‐reactive protein (mg/dl)	7	8	8	<10
Procalcitonin (ng/ml)	0.2	0.2	0.3	0.1–0.49
HBs‐ag	Negative	Negative	Negative	
HCV‐ag	Negative	Negative	Negative	
Tacrolimus level (ng/ml)	3.9	‐	‐	5–15
Cyclosporine level (ng/ml)	‐	‐	30	50–400

**FIGURE 1:**
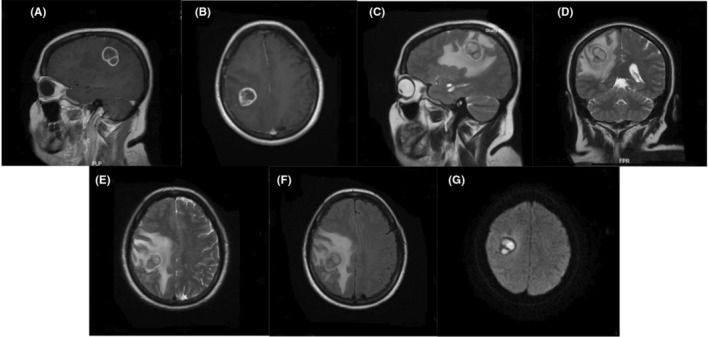
Mass with a calcified internal septum and ring enhancement in the right parietal lobe. (A) Sagittal T1‐Weighted, (B) Axial T1‐Weighted, (C) Sagittal T2‐Weighted, (D) Coronal T2‐Weighted, (E) Axial T2‐Weighted, (F) Axial T2‐weighted‐Fluid‐Attenuated Inversion Recovery (FLAIR), (G) Diffusion‐weighted MRI (DWI)

Physical examination showed normal findings. Laboratory analysis showed low hemoglobin (11.1 g/dl) (laboratory findings have been shown in Table 1). Ultrasonography exhibited the transplanted kidney in the right iliac fossa with a size of 75 × 140 × 75 mm (volume of 340 cc and larger than normal). Brain magnetic resonance imaging (MRI) revealed a mass with a calcified internal septum and ring enhancement in the right parietal lobe (Figure 1). The serum antibodies analysis for toxoplasmosis was negative. The histopathology study of the biopsy sample showed septate branching fungal hyphae suggestive of aspergillus fumigatus infection.

**FIGURE 2:**
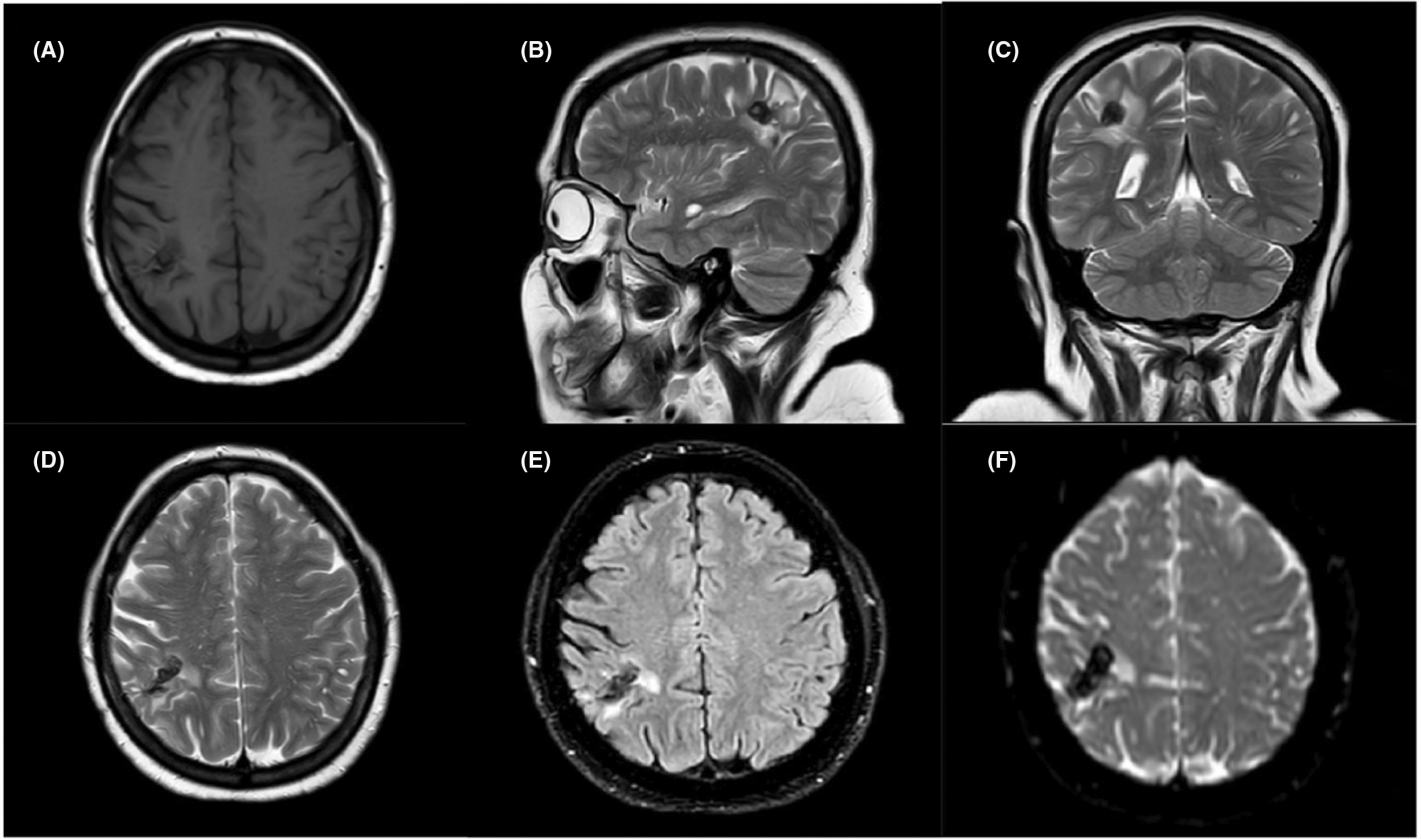
Encephalomalacia region with peripheral gliosis at the right parietal lobe due to previous surgery. (A) Axial T1‐Weighted, (B) Sagittal T2‐Weighted, (C) Coronal T2‐Weighted, (D) Axial T2‐Weighted, (E) Axial T2‐weighted‐Fluid‐Attenuated Inversion Recovery (FLAIR), (F) Diffusion‐weighted MRI (DWI)

First, it was decided to initiate the medical management for patient. The patient received voriconazole (400 mg, intravenous day one followed by 300 mg, oral for 14 days). According to the normal blood sugar, the insulin regimen was continued as it was before hospitalization. However, the symptoms (headache and other neurological symptoms) remained unchanged. Craniotomy was not performed due to the location of the lesion and immunosuppression state. Finally, the patient underwent neuronavigation‐guided surgery (stereotactic biopsy). It is worth mentioning that the antifungal therapy was not continued after surgery. The patient was discharged 2 weeks after brain surgery in good general condition. The Barthel index was 18/20 2 months after surgery. The patient was visited monthly for 1 year. Then, she was followed up every 3 months. Follow‐up of the patient for 4 years showed normal physical examination and laboratory findings. In addition, she had normal neurological functions during the follow‐up. It is worth mentioning that the Barthel index reached 20/20, indicating normal daily activities. Likewise, Brain MRI after 2 years showed an encephalomalacia region with peripheral gliosis at the right parietal lobe due to previous surgery (Figure 2). Due to severe immunosuppression induced by tacrolimus, which contributed to brain abscess, the tacrolimus was changed to cyclosporine.

## 3 DISCUSSION

We presented a case of an aspergillus brain abscess secondary to kidney transplantation in a 48‐year‐old woman with neurological symptoms. In immunosuppressed patients, aspergillus fumigatus, toxoplasma gondii, and nocardia asteroids are the most common causes of focal brain lesions.^6^ There are two mechanisms for CNS infection by aspergillus. First, spores enter the lungs through inhalation and spread hematogenous to CNS as angioinvasive aspergillosis. Second, aspergillus can spread directly to CNS through the paranasal sinuses, as invasive fungal rhinosinusitis.^7^ In our patient, the second mechanism is more likely due to normal pulmonary imaging findings, absence of pulmonary symptoms, and negative blood culture.

Hematogenous infections usually involve brain lobes and cause focal neurological deficits or non‐specific neurological symptoms such as headache, paresthesia, and altered mental status. Moreover, radiologic findings may exhibit ring enhancement with gross hemorrhage. However, they are not pathognomonic findings. Annular enhancement is more common in parenchymal involvement than meningeal aspergillosis on MRI.^8^, ^9^ Brain abscesses and granulomatous lesions are the most common pathological manifestations of brain aspergillosis. Pathogenesis usually depends on the location of the lesion and the host immune response. Infection in immunosuppressed patients (such as transplanted patients, as we saw in our patients) usually involves cerebral lobes (abscess with intact cyst wall). However, nodular granulomatous lesions and abscess wall rupture are probable.^10^ In addition, glucose and chloride levels may decrease in cerebrospinal fluid (CSF). Although these changes are not common, besides other signs and symptoms could help the early diagnosis. Positive CSF culture confirms the CNS aspergillosis.^11^ In this case, laboratory analysis of CSF did not show abnormal findings, which indicates that the normal CSF analysis does not rule out the CNS aspergillosis for sure.

The most important risk factor for invasive aspergillosis is immunosuppression, seen in prolonged neutropenia, hematologic malignancies, chemotherapy, corticosteroid consumption, and other immunosuppressive therapies such as biologic medications.^12^ Furthermore, diabetes—a major risk factor for invasive aspergillosis—may expose immunosuppressed patients to severe complications, as we saw in this patient.^13^ In this study, the patient was receiving the insulin for diabetes and due to normal blood sugar level, the same insulin regimen was continued during the hospitalization.

Voriconazole (intravenous/oral) is the first‐line and standard treatment for brain aspergillosis, and it showed more promising effects than other therapies such as amphotericin B. Liposomal amphotericin B could be a good primary alternative therapy. Lipid complex amphotericin B, caspofungin, and posaconazole are suggested in refractory cases or those who could not tolerate the first‐line drugs. Moreover, combination therapy of two antifungal drugs as first‐line treatment may be helpful. In the current study, despite the aforementioned drugs administration, desired results were not achieved. Immunosuppressant therapy discontinuation for those who were under long‐term immunosuppressive therapy may be needed. According to the multiple‐drug regimen of these patients, attention to drug interactions is necessary.^10^ However, it seems pharmacological treatment without surgical resection is not effective. Voriconazole and itraconazole should be continued after surgical removal of abscess and granuloma (to eliminate the residual aspergillosis not removed surgically). The combination of voriconazole and resection surgery could increase the chance of recovery up to 35%.^14^, ^15^ In this study, craniotomy was not performed due to the location of the lesion, concurrent morbidities, and immunosuppression state. We used neuronavigation guided surgery. However, we did not continue antifungal therapy after surgery. Neuronavigation facilitates brain minimally invasive surgeries and reduces morbidity in high‐risk patients. Wirtz et al.^16^ showed that neuronavigation‐guided surgery is associated with the lower residual tumor and more prolonged survival after brain glioblastoma resection surgery. Our patient can confirm the effectiveness of the neuronavigation‐guided surgery in immunosuppressed patients with brain lesions.

The mortality rate of invasive aspergillosis is about 50% or more. However, CNS invasive aspergillosis has a more unsatisfactory outcome and its mortality rate in immunosuppressed patients is near 100% and in an immunocompetent host is about 67%. Nevertheless, as we saw in this patient, early diagnosis, treatment with aggressive antifungal therapy (combination therapy), and neuronavigation‐guided surgery (or other surgical procedures) may improve the prognosis.^12^, ^15^

Moreover, Epstein et al.^17^ reported a 46‐year‐old man with underlying promyelocytic leukemia who developed an aspergillus brain abscess in the temporal lobe. In their study, the patient improved after receiving amphotericin B without surgery. Choudhury et al.^18^ reported a 50‐year‐old woman with diabetes who underwent liver transplantation and developed six hemorrhagic lesions due to aspergillosis ranging from 0.3 to 1.1 cm. Voriconazole and amphotericin B were administered, and the patient improved during the 6 months. However, Bao et al.^19^ described a 42‐year‐old man with a history of right parietal lobe tumorectomy and immunosuppressed state due to glucocorticoids administration and surgery‐induced trauma, who received the medical treatment without surgery and died 1.5 years later due to recurrent infections. In the same line, Tang et al.^20^ reported a 47‐year‐old man with alcoholic liver cirrhosis and multifocal aspergillosis brain lesions who received the amphotericin B and died after 9 days. Studies mentioned above showed that pharmacological treatment without surgical resection might be associated with contradictory outcomes, depends on the severity of disease and the clinical condition of patients. In the present study, due to the lack of response to pharmacological treatment and the location of abscess, the neuronavigation surgery was done and contributed to good outcome. This implies the importance of the severity of disease and clinical condition of patients in the selection of pharmacological treatment alone or in the combination of surgical resection.

In summary, we presented a case of aspergillus brain abscess in a 48‐year‐old woman with a history of kidney transplantation. This is a rare and fatal complication of immunosuppression. However, this case report highlights the important role of early diagnosis and aggressive antifungal therapy accompanied by neuronavigation‐guided surgery in increasing the chance of recovery. Follow‐up of the patient for 4 years shows the success of this approach.

## AUTHORS CONTRIBUTIONS

Seyed Parsa Eftekhar prepared the first draft of the paper. Seyed Parsa Eftekhar and Roghayeh Akbari revised and edited the first draft of the paper. All authors read the final draft of the manuscript and approved it.

## FUNDING INFORMATION

The authors declare that they have no competing financial interests.

## CONFLICT OF INTEREST

The authors declare no conflict of interest.

## CONSENT

Written informed consent was obtained from the patient to publish this report in accordance with the journal's patient consent policy.

## ETHICAL APPROVAL

This study was approved by the Ethics Committee of Babol University of Medical Sciences (Babol, Iran).

## Data Availability

The data supporting the findings of this study are available on request from the corresponding author and with permission from Babol University of Medical Sciences, Babol, Iran.
